# 11β hydroxysteroid dehydrogenase type 1 transgenic mesenchymal stem cells attenuate inflammation in models of sepsis

**DOI:** 10.3389/fbioe.2024.1422761

**Published:** 2024-07-05

**Authors:** Rahul Y. Mahida, Zhengqiang Yuan, Krishna K. Kolluri, Aaron Scott, Dhruv Parekh, Rowan S. Hardy, Michael A. Matthay, Gavin D. Perkins, Sam M. Janes, David R. Thickett

**Affiliations:** ^1^ Birmingham Acute Care Research Group, Institute of Inflammation and Ageing, University of Birmingham, Birmingham, United Kingdom; ^2^ School of Biomedical and Pharmaceutical Sciences, Guangdong University of Technology, Guangzhou, China; ^3^ Lungs for Living Research Centre, UCL Respiratory, University College London, London, United Kingdom; ^4^ Institute of Clinical Sciences, University of Birmingham, Birmingham, United Kingdom; ^5^ Cardiovascular Research Institute, Department of Medicine and Department of Anaesthesia, University of California San Francisco, San Francisco, CA, United States; ^6^ Warwick Medical School, University of Warwick, Warwick, United Kingdom

**Keywords:** mesenchymal stem cell, HSD-1, transfection, sepsis, macrophage

## Abstract

**Background:**

Human bone marrow mesenchymal stem cell (MSC) administration reduces inflammation in pre-clinical models of sepsis and sepsis-related lung injury, however clinical efficacy in patients has not yet been demonstrated. We previously showed that Alveolar Macrophage (AM) 11β-hydroxysteroid dehydrogenase type-1 (HSD-1) autocrine signalling is impaired in critically ill sepsis patients, which promotes inflammatory injury. Administration of transgenic MSCs (tMSCs) which overexpress HSD-1 may enhance the anti-inflammatory effects of local glucocorticoids and be more effective at reducing inflammation in sepsis than cellular therapy alone.

**Methods:**

MSCs were transfected using a recombinant lentiviral vector containing the HSD-1 and GPF transgenes under the control of a tetracycline promoter. Thin layer chromatography assessed HSD-1 reductase activity in tMSCs. Mesenchymal stem cell phenotype was assessed by flow cytometry and bi-lineage differentiation. HSD-1 tMSCs were co-cultured with LPS-stimulated monocyte-derived macrophages (MDMs) from healthy volunteers prior to assessment of pro-inflammatory cytokine release. HSD-1 tMSCs were administered intravenously to mice undergoing caecal ligation and puncture (CLP).

**Results:**

MSCs were transfected with an efficiency of 91.1%, and maintained an MSC phenotype. Functional HSD-1 activity was demonstrated in tMSCs, with predominant reductase cortisol activation (peak 8.23 pM/hour/100,000 cells). HSD-1 tMSC co-culture with LPS-stimulated MDMs suppressed TNFα and IL-6 release. Administration of transgene activated HSD-1 tMSCs in a murine model of CLP attenuated neutrophilic inflammation more effectively than transgene inactive tMSCs (medians 0.403 v 1.36 × 10^6^/ml, *p* = 0.033).

**Conclusion:**

The synergistic impact of HSD-1 transgene expression and MSC therapy attenuated neutrophilic inflammation in a mouse model of peritoneal sepsis more effectively than MSC therapy alone. Future studies investigating the anti-inflammatory capacity of HSD-1 tMSCs in models of sepsis-related direct lung injury and inflammatory diseases are required.

## Introduction

Sepsis is a potentially life-threatening multi-organ dysfunction caused by a dysregulated immune response to infection. A major cause for admission to Intensive Care Units (ICUs), sepsis has a global mortality rate of 22% with recent evidence showing rising incidence in the U.K. (Burki, 2018; Rudd et al., 2020). Acute Respiratory Distress Syndrome (ARDS) is a hyper-inflammatory pulmonary disorder which can occur secondary to sepsis; development of ARDS requires initiation of mechanical ventilation, and raises the mortality rate to 40% (Bellani et al., 2016). The mainstay of sepsis therapy includes antimicrobial therapy and supportive management (Singer et al., 2016). Despite improvements over recent decades (Kaukonen et al., 2014), sepsis remains a major cause of inpatient mortality, indicating an unmet clinical need for novel therapeutic interventions. Numerous studies have shown that bone marrow mesenchymal stem cells (MSCs) can attenuate inflammation and promote recovery in pre-clinical models of sepsis, indicating therapeutic potential.

MSCs have been shown to be efficacious in small and large animal models of sepsis (Xu et al., 2007; Gonzalez-Rey et al., 2009; Nemeth et al., 2009; Mei et al., 2010; Krasnodembskaya et al., 2012) and sepsis-related lung injury (Cárdenes et al., 2013; Asmussen et al., 2014; Rojas et al., 2014; McIntyre et al., 2016). Phase 1 and 2a clinical trials in patients with sepsis and ARDS have shown that MSC administration is safe, feasible and well-tolerated, with no adverse effects (Galstian et al., 2015; Wilson et al., 2015; Matthay et al., 2019; Gorman et al., 2021). However, phase 3 clinical trials are still required to determine MSC efficacy in patients. There are multiple factors which support the use of MSCs as a clinical therapy for sepsis: Due to their low expression of major histocompatibility antigens, allogenic MSCs can be administered without inducing an immune response (Patel et al., 2008). MSCs can be expanded *in vitro*, and retain their efficacy following cryopreservation (Devaney et al., 2015; Yuan et al., 2016). The main mechanisms by which MSC attenuate inflammatory injury are the promotion of epithelial and endothelial repair, and modulation of immune function to increase bacterial clearance and aid resolution of inflammation (Laffey and Matthay, 2017). These mechanisms can be initiated by either cell-to-cell contact or release of paracrine factors. However, studies have also shown that MSCs lose their intrinsic anti-inflammatory capabilities with repeated *in vitro* passage (Choi et al., 2010). Therefore, the expansion required to generate sufficient MSCs for clinical therapy may also decrease their anti-inflammatory functions, thereby limiting their therapeutic potential.

A key mechanisms by which MSCs induce their anti-inflammatory effects is via modulation of macrophage function. MSCs produce prostaglandin E2, which stimulates macrophages to secrete the anti-inflammatory cytokine IL-10, but also enhances their bacterial killing ability (Gupta et al., 2007; Xu et al., 2007; Nemeth et al., 2009; Rabani et al., 2018). Release of Lipoxin A4 by MSCs also enhances macrophage phagocytosis (Maderna et al., 2010). Production of IL-1 receptor antagonist by MSCs downregulates macrophage secretion of the pro-inflammatory cytokine TNFα (Ortiz et al., 2007). MSCs can transfer mitochondria to macrophages either via tunnelling nanotubules requiring direct contact (Jackson et al., 2016), or remotely via extracellular vesicles (Morrison et al., 2017). By these mechanisms, MSCs induce a modified M2 macrophage phenotype, which has pro-resolving characteristics, but also increased phagocytic activity against bacteria (Kim and Hematti, 2009; Ionescu et al., 2012; Krasnodembskaya et al., 2012; Morrison et al., 2017). This ensures more effective bacterial clearance, whilst minimising tissue injury.

Our work has demonstrated that alveolar macrophage dysfunction may play a role in the pathogenesis of sepsis-related ARDS (Mahida et al., 2021a). Alveolar macrophage efferocytosis (clearance of apoptotic cells) is impaired in patients with sepsis-related ARDS, and is associated with increased 30-day mortality in ICU sepsis patients (Mahida et al., 2021a). Macrophage efferocytosis is a pro-resolving function associated with the suppression of inflammation (Fadok et al., 1998; Lee et al., 2012). We also investigated the role of alveolar macrophage glucocorticoid metabolism in patients with sepsis-related ARDS. 11β-hydroxysteroid dehydrogenase type-1 (HSD-1) acts as a reductase to convert inactive cortisone to active cortisol, thus amplifying glucocorticoid action in peripheral tissues (Chapman et al., 2013); expression of this enzyme is induced on differentiation of monocytes into tissue macrophages (Thieringer et al., 2001). Elevated HSD-1 activity and local cortisol activation promote macrophage polarisation towards an anti-inflammatory phenotype and enhance efferocytosis of apoptotic cells (Liu et al., 1999; Gilmour et al., 2006; Zizzo et al., 2012). We showed that alveolar macrophages from ARDS patients have impaired HSD-1 autocrine signalling, which renders alveolar macrophages insensitive to the anti-inflammatory effects of local glucocorticoids (Mahida et al., 2023). This impaired macrophage HSD-1 signalling contributes to the decreased efferocytosis, epithelial injury and increased mortality seen in sepsis-related ARDS (Mahida et al., 2023). Models of peritoneal sepsis and inflammatory lung injury in HSD-1 knockout mice demonstrated increased alveolar neutrophil infiltration, apoptotic neutrophil accumulation, and epithelial injury compared to wild type mice (Mahida et al., 2023).

Exogenous corticosteroid therapy may not improve clinical outcomes in sepsis patients due to the impairment of tissue macrophage HSD-1 autocrine signalling (Rochwerg et al., 2018). Thus, strategies to upregulate macrophage HSD-1 reductase activity could facilitate both endogenous and exogenous glucocorticoids to exert their anti-inflammatory effects locally. This may restore macrophage efferocytosis, thereby reducing secondary necrosis of apoptotic neutrophils and attenuating alveolar inflammation. Strategies to achieve this include HSD-1 gene therapy targeted to sites of inflammation.

The characteristics of MSCs which support their use as clinical therapy also make them ideal vectors for gene therapy. Use of transgenic MSCs (tMSCs) in murine models of sepsis-related lung injury have shown that the synergistic anti-inflammatory action of combined cellular and gene therapy can be superior to that of cellular therapy alone (Mei et al., 2007; Xu et al., 2008; Wang et al., 2018). Transgenes delivered by tMSCs in these models have included Interleukin-10 (Wang et al., 2018) and Angiopoetin-1 (Mei et al., 2007; Xu et al., 2008). We postulated that administration of tMSCs expressing the HSD-1 transgene in murine models of sepsis would elevate tissue cortisol levels. This, in combination with the innate immuno-modulatory abilities of the MSCs, would act synergistically to enhance macrophage efferocytosis and support the resolution of inflammation in sepsis.

## Materials and methods

### Creation of HSD-1 transgenic MSCs

#### Creation of recombinant HSD-1 lentiviral plasmid

A lentiviral plasmid (pRRL-cPPT-hPGK-mcs-WPRE) including the Tet-on system elements, had the reporter gene MuSEAP excised and replaced with the IRES-eGFP sequence (Internal Ribosome Entry Site–Enhanced Green Fluorescent Protein from pENTR1A) (Loebinger et al., 2009). This modified lentiviral plasmid was used as the backbone for the incorporation of human HSD-1 DNA.

Human HSD-1 cDNA (amino acids 1–293, Origene) was amplified and restriction sites for MluI and BstB1 were introduced using polymerase chain reaction (PCR). Forward amplification primer sequence (Invitrogen) was CGT​ACG​CGT​GCC​ACC​ATG​GCT​TTT​ATG​AAA​AAA​TAT​CTC​CTC​CC. Reverse amplification primer sequence was GTC​GTT​CGA​ACT​ACT​TGT​TTA​TGA​ATC​TGT​CC. The HSD-1 PCR product and the lentiviral backbone plasmid were then both sequentially digested with MluI then BstB1 (New England Biolabs). The digested HSD-1 insert was ligated into the digested lentivirus plasmid vector next to the IRES-eGFP, using T4 DNA ligase (New England Biolabs) to create the recombinant plasmid. Recombinant plasmids were amplified in DH5α *E. coli* (*Escherichia coli,* ThermoFisher) grown in LB broth supplemented with 100 μg/ml ampicillin, and purified using HiSpeed Plasmid Maxip kits (Qiagen). Recombinant plasmid constructs were confirmed by DNA sequence analysis (Source Bioscience).

##### Production of recombinant HSD-1 lentivirus

Human Embryonic Kidney 293T cells (HEK 293T, Sigma) were cultured in T175 flasks with Dulbecco’s Modified Eagle’s Medium (DMEM, Sigma) including 10% Foetal Bovine Serum (FBS), at 37°C and 5% CO_2_ until reaching 80%–90% confluence. HEK 293T cells were transfected using a second-generation packaging system: 2 ml of a 150 nM NaCl solution containing 80 μL linear polyethylenimine (jetPEI transfection reagent, Polyplus transfection), 20 μg recombinant HSD-1 plasmid, 13 μg packaging plasmid pCMV-dR8.74, and 7 μg envelope plasmid pMD.G2 were added to each flask. Media containing recombinant lentivirus was collected at 48 and 72 h post-transfection. Supernatant media was centrifuged at 300 *g* for 10 min at 4°C, then passed through a 0.45 μm filter. Recombinant lentivirus within the media was then concentrated by ultracentrifugation (SW28 rotor, Optima LE80K Ultracentrifuge, Beckman) at 52,000g and 4°C for 2 h. Supernatants were discarded and the lentiviral pellet re-suspended in serum-free DMEM, prior to storage at −80°C.

#### Determination of viral titre

HEK 293T cells were treated with 8 μg/ml hexamethadine bromide (Polybrene, Sigma) transfection reagent, and serial dilutions of recombinant HSD-1 lentivirus (1:100 to 1:10,000). After 24 h 10 μg/ml doxycycline was added, which acted as the transcriptional activator for the HSD-1 and GFP transgenes. After a further 24 h, cells were harvested and assessing for GFP expression using flow cytometry (LSR Fortessa X-20, BD Biosciences). The viral titre was calculated (Giry-Laterriere et al., 2011).

### Transfection of MSCs

Human bone-marrow derived MSCs from 4 young adult donors were purchased from the Institute of Regenerative Medicine at Texas A&M College of Medicine, USA. Donors were designated: 8011 (24-year-old female), 8004 (22 year old male), 8013 (22-year-old male), and 7083 (24 year old male). MSCs were cultured in α-Minimal Essential Media (αMEM, ThermoFisher) containing 16% FBS; cell numbers were counted at each passage (Supplementary Figure S1A. MSC secretion of pro-resolving mediators including angiopoetin-1, vascular endothelial growth factor (VEGF) and transforming growth factor-β (TGF-β) into conditional media was assessed by ELISA (DuoSet^®^, R&D systems) as per manufacturer’s instructions (Supplementary Figures S1C, D).

MSCs from donor 8011 were chosen to be transfected, as MSCs from this donor had shown the most rapid growth *in vitro* (Supplementary Figure S1A). Prior to and following transfection, these MSCs were cultured using tetracycline-free FBS (lot 42G9273K, ThermoFisher Scientific), to prevent uncontrolled expression of GFP and HSD-1 transgenes. MSCs from this donor at passage 2 were plated at 100,000 per T75 flask and cultured overnight at 37°C and 5% CO_2_ to allow adherence. Media was removed and replaced with αMEM containing 8 μg/ml polybrene and 200,000 lentiviral transduction units. MSCs were transfected with a multiplicity of infection (MOI) of 2 virus particles for each cell.

#### Assessment of HSD-1 transgene expression

The tMSCs were cultured with 10 μg/ml doxycycline for 48 h, before being fixed in 4% paraformaldehyde, then made permeable by incubating in saponin buffer (PBS/10% FBS/0.1% saponin). Cells were incubated with primary rabbit anti-human HSD-1 monoclonal antibody (ab157223, Abcam) at 1:100 dilution, then secondary donkey anti-rabbit IgG H&L antibody Alexa-fluor^®^ 555 (ab150074, Abcam) at 1:500 dilution, prior to analysis on flow cytometry (LSR Fortessa X-20).

Expression of HSD-1 protein in tMSCs was also assessed by Western blot. 1 × 10^6^ non-transfected MSCs and tMSCs (previously cultured with 10 μg/ml doxycycline) were harvested. Cells were lysed using RIPA buffer (Cell Signalling) then protein concentrations of cell lysates were calculated using a bicinchoninic acid assay (ThermoFisher). Samples were denatured at 70°C and resolved on a Bolt 4%–12% Bis-Tris-Plus gel (ThermoFisher) and MES SDS (2-(*N*-morpholino)ethanesulfonic acid, sodium dodecyl sulfate) running buffer at 15 μg or 22 μg protein/well. Samples were transferred onto a membrane using Novex iBlot transfer system (ThermoFisher). The membrane was incubated with primary rabbit anti-HSD-1 monoclonal antibody (ab157223, Abcam) at 1:10,000 dilution, then Goat polyclonal anti-Rabbit HRP-conjugated antibody (P0448, Dako) at 1:2000 dilution, then Horseradish Peroxidase substrate (HRP substrate, Merck Millipore) prior to detection (Image Quant LAS 4000, GE). To assess total protein loading the membrane was stripped, labelled with anti- β-tubulin rabbit antibody (9F3, Cell Signalling) at 1:1000, then Goat anti-Rabbit HRP-conjugated antibody at 1:2000, then HRP substrate prior to detection (ChemiDoc, Bio-Rad).

#### Adipogenic and osteogenic differentiation of MSCs

Bi-lineage adipogenic and osteogenic differentiation kits (StemPro) were used; protocols were as per manufacturer’s guidance. MCSs cultured in αMEM/16% FBS acted as negative control. For adipocyte detection, MSCs were stained with a 0.2% stock solution of Oil Red O (Sigma-Aldrich) which is taken up by lipid inclusion vacuoles. For osteocyte detection, MSCs were stained with a 2% solution of Alizarin Red S (Sigma-Aldrich) which is taken up by calcium deposits. Following staining, photographs were taken via a microscope (Zeiss AxioVert A1).

#### Flow cytometry assessment of MSC surface markers

HSD-1 tMSCs and non-transfected MSCs were labelled with the following anti-human antibodies or their isotype controls: CD105-BV786, CD73-BV421, CD90-PE (positive cocktail, all purchased from BD Biosciences) or CD14-APC, CD19-APC, CD34-APC, CD45-APC, and HLADR-APC (negative cocktail, all purchased from ThermoFisher). Surface marker expression was assessed by flow cytometry (LSR Fortessa X-20). Compensation and analysis were performed using FACSDiva Software (BD Biosciences).

#### Thin layer chromatography assay to assess HSD-1 activity in MSCs and mouse lung tissue

This assay was developed and has previously been validated by the Institute for Metabolism and Systems Research at the University of Birmingham (Bujalska et al., 1999; Bujalska et al., 2002).

MSCs and HSD-1 tMSCs were pre-treated with either vehicle control, 10 μg/ml doxycycline, 50 ng/ml tumour necrosis factor-α (TNF-α, Peprotech) or 100 ng/ml Lipopolysaccaride (LPS, Sigma) for 48 h. To inhibit HSD-1 activity, cells were incubated with 10^−7^ M glycyrrhetinic acid (Sigma) for 24 h. HSD-1 reductase activity (conversion of cortisone to cortisol) was determined in adherent cultures containing 250,000 cells incubated in αMEM medium with cortisone (100 nmol/L) along with tracer amounts of tritiated cortisone (Perkin Elmer) at 37°C and 5% CO_2_ for 6–12 h (Bujalska et al., 1999; Bujalska et al., 2002). Steroids were extracted in dichloromethane and separated by thin‐layer chromatography with ethanol/chloroform (8:92) as the mobile phase. Thin‐layer chromatography plates were analysed with a Bioscan imager (Bioscan, Washington, DC, USA), and the fractional conversion of steroids was calculated. HSD-1 activity is expressed in pM/hr/million cells.

An equivalent methodology was utilised to measure HSD-1 oxo-reductase activity in murine *ex-vivo* lung tissue as previously described (Fenton et al., 2021). Murine lung tissue was incubated with 100 nmol/L of 11-dehydrocorticosterone (11-DHC) and tritiated [3H] tracer. As above, steroids were extracted and separated before steroid conversion was measured using a Bioscan imager and fractional conversion calculated. Experiments were performed in triplicate, and enzymatic activity is reported as pmol product per mg of tissue per hour.

### Clinical studies

#### Patient recruitment

The AM-ARDS study was conducted at the ICU of Queen Elizabeth Hospital Birmingham, U.K. from December 2016 to January 2019. Ethical approval was obtained to recruit invasively ventilated adult sepsis patients, with and without ARDS (REC 16/WA/0169). Sepsis was defined according to Sepsis-3 criteria (D'Alessio et al., 2016). Patients who fulfilled the Berlin criteria (The, ADTF, 2012) within the previous 48 h were classified as having ARDS; those without ARDS were defined as controls. Exclusion criteria included imminent treatment withdrawal, steroid therapy prior to admission, abnormal clotting precluding bronchoscopy, and clinically relevant immunosuppression. Samples were collected within 48 h of initiation of mechanical ventilation. Patients were unable to give informed consent due to alterations in conscious level caused by illness and therapeutic sedation. Therefore, their next of kin were requested to give assent for the patient to be recruited into the study.

Adult patients due to undergo lung lobectomy as part of their clinical treatment plan for malignancy at Birmingham Heartlands Hospital from September 2017 to July 2019 were also recruited (REC 17/WM/0272). Recruited patients were never-smokers or long-term ex-smokers (quit >5 years), with normal spirometry and without airways disease. No patient received chemotherapy prior to surgery. Following lobectomy, lung tissue resection samples surplus to histopathological requirements were collected.

#### Human broncho-alveolar fluid collection

Bronchoscopy and BAL fluid collection was performed on sedated, mechanically ventilated patients using a standardised protocol, within 48 h of initiation of mechanical ventilation. Patients were ventilated using 100% inspired oxygen for 5 min prior to bronchoscopy. An Olympus LF-TP fiberoptic scope (Olympus-Keymed) was inserted through the patient’s endo-tracheal tube, and the tip was wedged into a sub-segmental bronchus of the lingula or right middle lobe. Two 50 mL aliquots of sterile 0.9% saline at room temperature were instilled as a lavage, and the BAL fluid was aspirated. BAL fluid was filtered through sterile gauze to remove mucus. Differential cell count was performed using cytospin and Diff-Quik labelling (Gentaur Europe). The filtered BAL was then centrifuged at 560 *g* for 10 min at 4°C. Acellular BAL supernatant was aspirated and stored at −80°C. BAL samples from 16 patients with sepsis-related ARDS were pooled, characterised for cytokine content as previously described (Mahida et al., 2021b) and used to treat primary alveolar macrophages from lung tissue samples.

#### Alveolar macrophage isolation from lung tissue samples

Non-affected, macroscopically normal lung tissue samples were perfused with 0.15M saline via pressure bag by inserting a needle (21-gauge) in bronchioles, as previously described (Scott et al., 2018; Mahida et al., 2021b; Mahida et al., 2023). Cells were pelleted from the lavage fluid by centrifugation at 500 *g* for 5 min. Mononuclear cells were then separated by gradient centrifugation using Lymphoprep (StemCell Technologies), according to the manufacturer’s instructions. Mononuclear cells were cultured in RPMI-1640 media supplemented with 10% FBS, 100U/mL penicillin, 100ug/mL streptomycin and 2 mM L-glutamine (Sigma-Aldrich) at 37°C and 5% CO_2_; media was changed after 24 h to remove non-adherent cells (O'Kane et al., 2009; Davies and Gordon, 2005). Flow cytometric staining with CD68 (APC-conjugated mouse anti-human CD68, clone FA-11, Biolegend U.K.) was undertaken to confirm a pure population of alveolar macrophages (AMs). If there was greater than 2% contamination of non-AM cells including interstitial macrophages, the sample was not utilised.

#### Isolation of human monocyte-derived macrophages

Peripheral blood mononuclear cells were isolated from the whole blood of healthy volunteers (REC 20/WA/0092) using a Lymphoprep™ (StemCell) density gradient, then incubated in RPMI-1640 including 10% FBS, at 37°C and 5% CO_2_ for 2 h. Non-adherent cells were removed by washing. Monocyte-derived-macrophages (MDMs) were then derived by incubating the adhered monocytes with complete media containing 10% human serum and 1 ng/ml granulocyte-macrophage colony stimulating factor (GM-CSF). After 6 days of culture, the MDMs were then utilised in co-culture assays. Purity of MDMs was >85%.

#### Direct and transwell MSC–macrophage co-culture assays

500,000 MDMs were co-cultured with 1.25 × 10^4^ MSCs or HSD-1 tMSCs at passage 4 in serum-free RPMI 1640, giving a 1:4 ratio. This ratio was chosen to replicate the predicted ratio of MSCs to macrophages in the lungs of ARDS patients undergoing clinical trials of MSC therapy (Ochs et al., 2004; Zheng et al., 2014; Wilson et al., 2015). Wells were treated with 200 ng/ml Ultra-pure Lipopolysaccaride (LPS, Invivogen). Exogenous cortisone and cortisol (Sigma-Aldrich) were added at 10^−7^ M; this is the physiological circulating concentration of cortisone (Seckl and Walker, 2001). Cells were cultured for 24 h at 37°C and 5% CO_2_, before conditioned media was aspirated from wells, centrifuged at 500 *g*, and frozen at −20°C. Human ELISA DuoSet kits (R&D Systems) measured concentrations of TNFα, and interleukin-6 (IL-6) in conditioned media as per manufacturer’s instructions.

Following isolation, AMs were cultured at 2.5 × 10^5^ per well in 24-well plates and rested for 24 h. To elicit functional changes associated with ARDS, the AMs were treated with this 50% ARDS BAL mixture. As a vehicle control treatment, AMs were also treated with a 1:1 mixture of 0.9% Saline (Baxter, UK) and RPMI-1640 including 10% FBS. To assess the impact of HSD-1 transgenic mesenchymal stem cells (tMSCs) on restoring AM function/phenotype after treatment with 50% ARDS BAL, a 6 h Transwell co-culture of passage 4 tMSCs with AMs was performed between removal of ARDS BAL and efferocytosis or phenotyping assays. After 24 h treatment with 50% ARDS BAL fluid or saline, AMs were washed with PBS and media changed to serum-free RPMI. 10^−7^ M exogenous cortisone (Sigma-Aldrich) was added to all wells. Transwell^®^ permeable inserts containing a 0.4 μm polycarbonate microporous membrane (Costar, Corning) were added to each well. 6.25 × 10^4^ transgene-activated tMSCs, transgene-inactive tMSCs or no cells in 100 µL serum-free RPMI were added to the upper compartment. The ratio of AMs to tMSCs was 4:1. Co-cultures were incubated for 6 h at 37°C and 5% CO_2_. The basis of the 6h co-culture duration was twofold: Firstly, HSD-1 functional assays on HSD-1 tMSCs revealed that 6 h allowed 100,000 tMSCs to convert ∼50% of the available cortisone into cortisol. Secondly, previous murine studies have found that MSC numbers in the lung halved between 1 and 3 h post-infusion (Rochefort et al., 2005). To recapitulate the duration that tMSCs would be present in the lungs if used as an intravenous clinical therapy, a co-culture duration of 6 h was used. After 6 h, the transwell inserts were removed, and the AM were washed in PBS prior to assessment of efferocytosis and phenotype as described below.

#### Alveolar macrophage efferocytosis assay

This protocol was performed as previously described (Mahida et al., 2021b). Briefly, neutrophils were isolated from the blood of healthy volunteers using Percoll density centrifugation (Jepsen and Skottun, 1982; Sapey et al., 2019). Neutrophils were suspended in a 5 μM solution of CellTracker™ Deep Red fluorescent dye (ThermoFisher) at 4 × 10^6^/ml, then incubated for 30 min at 37°C. Stained neutrophils were centrifuged at 1500 *g* for 5 min then incubated in serum-free RPMI at 37°C and 5% CO_2_ for 24 h to induce apoptosis. As negative control, 5 μg/ml Cytochalasin D (Sigma-Aldrich) was added to AMs for 30 min to inhibit actin filament polymerization. Stained apoptotic neutrophils (ANs) were added to AMs at a 4:1 ratio prior to incubation for 2 h at 37°C. Media was removed and wells washed twice with ice-cold PBS. Cells were harvested prior to acquisition using an Accuri C6 flow cytometer (BD Biosciences). Background fluorescence from negative controls was subtracted from the percentage of APC^+^ AMs in other experimental conditions, to give a corrected net efferocytosis index representative of neutrophil engulfment.

#### Flow cytometric assessment of AM surface markers

AMs were labelled with the following anti-human antibodies or their isotype controls: CD206-APC, CD80-PE, CD163-FITC, Mer-APC, and SIRPα-FITC (see Supplementary Table S1). Surface marker expression was assessed by an Accuri C6 flow cytometer and software (BD Biosciences). AM population was gated on forward and side-scatter plot. The median fluorescence intensity (MFI) in relevant channels from isotype control AMs was subtracted from the MFIs of stained AMs, to give the net MFI for each antibody fluorophore. Results presented as fold change corrected MFI, as a measure of change in cell surface expression, compared to vehicle control (50% saline).

### Murine studies

#### Mice

All procedures were performed in compliance with UK law under the Animal [Scientific Procedures] Act 1986. The UK Home Office project licence code was PAAB1C3B2. The 3Rs principles (Reduction, Replacement and Refinement) guided the design and methodology of our animal studies. Male Wild Type (WT) C57BL/6 mice were obtained from Harlan UK Limited, Oxford, UK and maintained at the Biomedical Services Unit, University of Birmingham, UK. A colony of 11β-Hydroxysteroid Dehydrogenase Type 1 Knockout (HSD-1 KO) C57BL/6J mice was also maintained at BMSU. The breeding pairs for this colony were a gift from Professor Gareth Lavery, University of Birmingham (Larner et al., 2016; Doig et al., 2017).

#### Caecal ligation and puncture mouse model

Caecal ligation and puncture was performed on WT mice aged 8–12 weeks as previously described (Parekh et al., 2017). Briefly, mice were anaesthetised with 5% isoflurane gas in oxygen delivered at 1.5 L/min for induction, then at 1%–3% isoflurane for maintenance anaesthesia. Midline laparotomy was performed followed by exposure of the caecum, ligation of the lower 30% with 2.0 nylon suture (Ethicon, UK) and single puncture of the ligated caecum with a 19G microlance needle (BD, UK). A small amount of faeces was expressed by compressing the ligated caecum with forceps prior to being placed back into the abdomen and closed. Mice were euthanised at 3 h post-surgery. Cardiac puncture was performed and blood was centrifuged at 13,500 rpm in a micro-centrifuge for 10 min. Serum was aspirated and stored at −40°C. Samples of peritoneal lavage fluid (PLF) were collected immediately post-mortem by instilling 1 mL of PBS/1% EDTA into both upper quadrants of the abdomen and subsequently aspirating from both lower quadrants.

#### Analysis of murine peritoneal lavage fluid

PLF was centrifuged at 400 *g* for 10 min; supernatants were aspirated and stored at −80°C. PLF cell pellets were incubated on ice in 2% BSA and 10% murine serum (Sigma-Aldrich) to block non-specific Fc receptor binding. PLF cell pellets were assessed for cellular inflammation and apoptotic cell number by flow cytometry (LSR Fortessa X-20) using fluorophore-conjugated antibodies (see Supplementary Table S2). Neutrophils were defined as CD11c^−^CD11b^+^Gr1^+^F4/80^-^, monocytes as CD11c^+^CD11b^+^, and F4/80^+^ as macrophages. Apoptosis was analysed as FITC - Annexin V and SyTOX Blue (Invitrogen) double positive populations. See Supplementary Figure S2 for the gating strategy.

PLF was diluted serially and incubated at 37°C in pre-prepared Lysogeny broth (LB-Lennox, Merck) agar plates for 24 h; bacterial colony forming units (CFU) were then counted and CFU/ml calculated from the original dilutions.

Murine serum and PLF inflammatory cytokines including monocyte chemoattractant protein-1 (MCP-1), macrophage inflammatory protein (MIP)-1α, MIP-1β, MIP-2, KC, IL-1β, IL-6, IL-10, TNFα and vascular endothelial growth factor (VEGF) were measured by a Luminex^®^ screening assay (R&D systems) as per the manufacturer’s protocols.

#### Statistical analysis

Data was analysed using Prism 9 software (GraphPad). Normality of data was assessed using the D’Agostino and Pearson test. Differences between two non-parametric data were assessed using Mann-Whitney tests. Differences between three or more non-parametric data sets were assessed using the Kruskal–Wallis one-way analysis of variance (ANOVA) and Dunn’s multiple comparison tests. Differences between three or more paired non-parametric data sets were assessed using the Friedman one-way analysis of variance (ANOVA) and Dunn’s multiple comparison tests. Differences between three or more paired parametric data sets were using two-way repeated measures ANOVA. Two-tailed *p*-values of <0.05 were considered as significant. Results from non-parametric data are shown as median and interquartile range. Results from parametric data are shown as mean and standard deviation.

## Results

### Creation of HSD-1 transgenic mesenchymal stem cells

**FIGURE 1:**
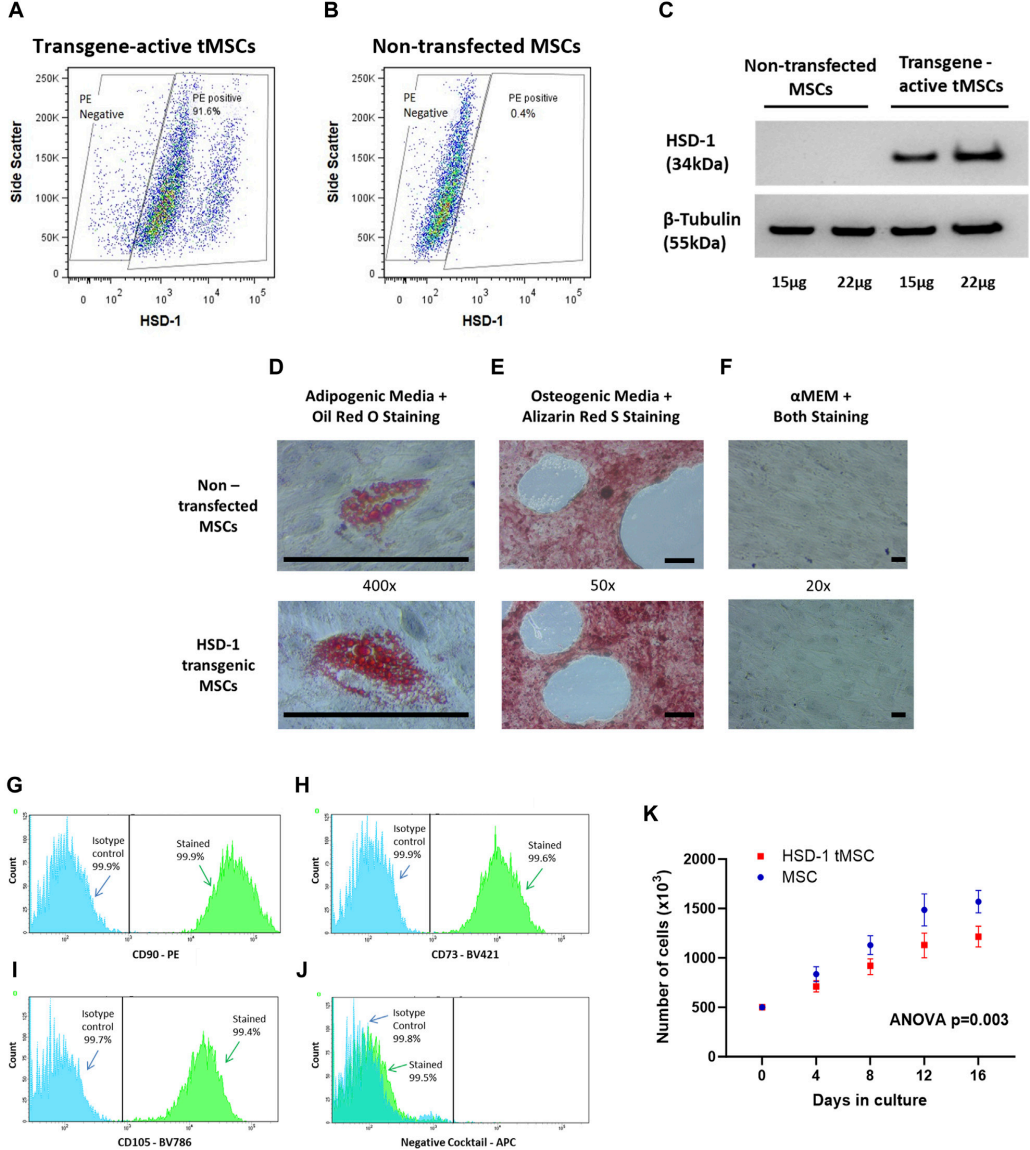
Creation and phenotypic assessment of HSD-1 transgenic MSCs (tMSCs). **(A, B)** Representative flow cytometry plots showing HSD-1 staining in transgene-activated tMSCs and non-transfected MSCs. **(C)** Representative Western blot shown. Protein from non-transfected MSCs and transgene-activated tMSCs was labelled for HSD-1; the predicted band size is 38 kDa. The membrane was stripped and re-labelled for beta-tubulin as a loading control. **(D–F)**: All cells shown are at passage 4. Non-transfected MSCs and tMSCs were successfully differentiated down **(D)** adipogenic and **(E)** osteogenic lineages. **(F)** When cultured in αMEM including 16% FBS (control), both MSCs and tMSCs showed no signs of lineage differentiation on dual staining. Scale bar ∼100 µm. **(G–I)** Overlaid fluorescence histograms of HSD-1 tMSCs labelled with antibodies against MSC positive markers (CD90, CD73 and CD105) or isotype controls. **(J)** The negative staining cocktail contained APC-conjugated antibodies against CD14, CD19, CD34, CD45, and HLA-DR. **(K)**: MSCs and HSD-1 tMSCs at passage 4 were plated at 500,000 cells per flask and cultured *in vitro*. Cells numbers were counted every 4 days up to 16 days. Statistical analysis by repeated-measures ANOVA, n = 4 for both groups.

The *in vitro* proliferative capacity of human bone-marrow derived MSCs from 4 donors was assessed (Supplementary Figure S1A). MSCs from a 24 year-old female (donor 8011) were chosen to be transfected as MSCs from this donor had shown the most rapid growth *in vitro*. MSCs were transfected with recombinant HSD-1 lentivirus, using a multiplicity of infection (MOI) of 2. Following transfection and culture with 10 μg/mL doxycycline to activate the GFP and HSD-1 transgenes, tMSCs were visualised under fluorescence microscopy to assess for GFP expression. Transgene-activated tMSCs were stained intracellularly for HSD-1 to assess for transfection efficiency, which was found to be 90.1% on flow cytometry (Figures 1A, B). HSD-1 protein was expressed in transgene-active tMSCs, and absent in non-transfected MSCs (Figure 1C, complete Western blot gel in Supplementary Figure S3). These findings indicated that the transfection process had been successful and that tMSCs were able to generate HSD-1 protein when cultured with doxycycline (transcriptional activator).

To show that HSD-1 tMSCs maintain a stem cell phenotype, we assessed differentiation capacity and surface marker expression as per the International Society for Cellular Therapy (ISCT) criteria (Dominici et al., 2006). Adipogenic and osteogenic differentiation experiments showed that tMSCs retain bi-lineage differentiation capacity (Figures 1D–F). Phenotypic assessment revealed HSD-1 tMSCs expressed CD90, CD105 and CD73 (Figures 1G–I). HSD-1 tMSCs also lacked expression of CD14, CD19, CD34, CD45 and HLA-DR (Figure 1J). Therefore, HSD-1 tMSCs retain the MSC surface marker configuration as per ISCT criteria. However, HSD-1 tMSCs proliferate at a slower rate *in vitro* compared to non-transfected MSCs (Figure 1K, ANOVA *p* = 0.003); the growth rate for both appears to plateau after 16 days.

### Functional assessment of the HSD-1 transgene in tMSCs

**FIGURE 2:**
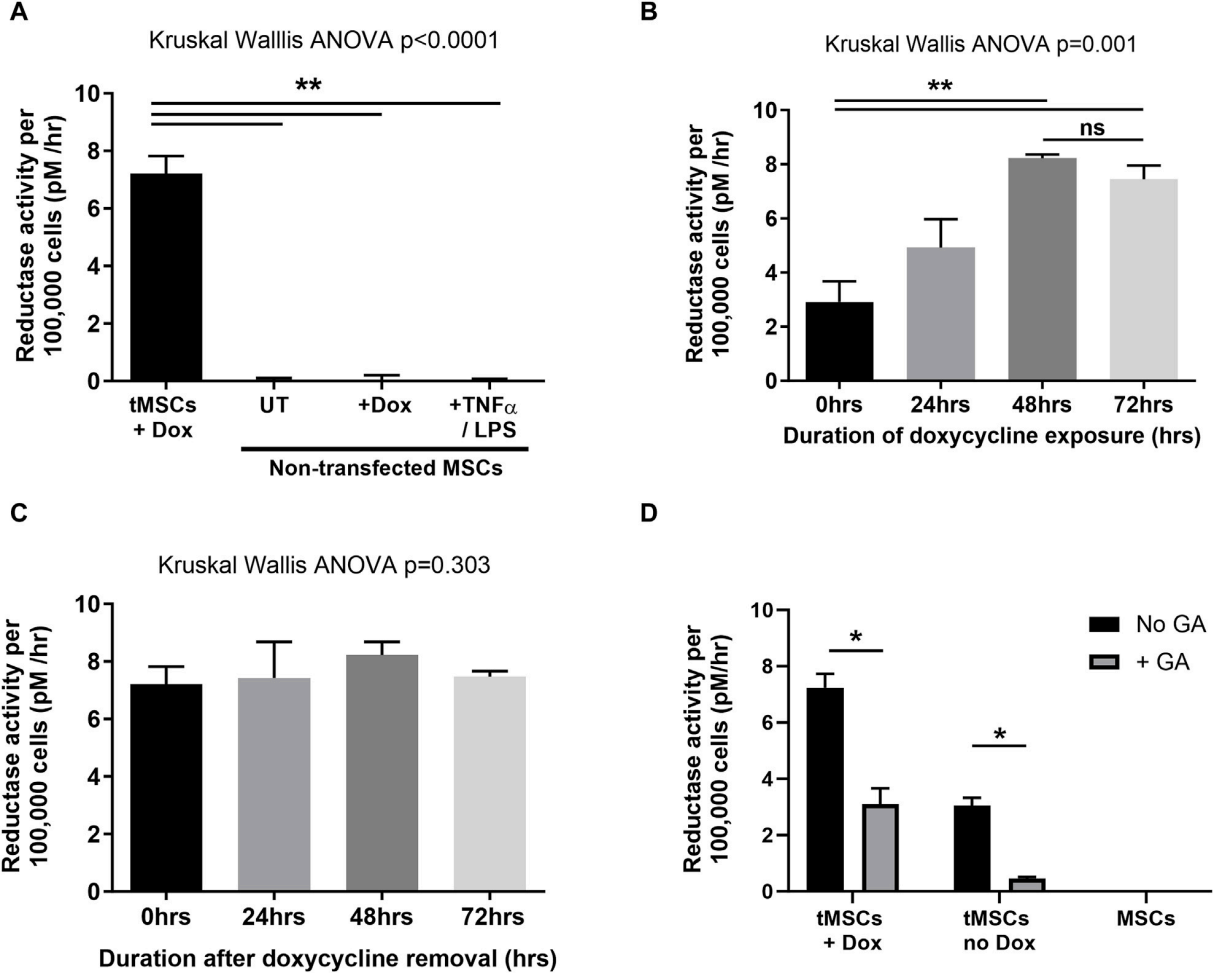
Assessment of HSD-1 functional activity in tMSCs. Data are shown as median and inter-quartile range, with n ≥ 4 for all conditions. Statistical analysis by Kruskal–Wallis ANOVA and Dunn’s multiple comparison test. **(A)** Impact of varying treatments on the HSD-1 reductase activity of non-transfected MSCs. **(B)** Reductase activity of HSD-1 tMSCs following differing durations of doxycycline (transcriptional activator) treatment. **(C)** HSD-1 tMSCs which had previously been exposed to doxycycline for 48 h, were then cultured in doxycycline-free media for differing durations prior to HSD-1 functional assay. **(D)** Impact of glycyrrhetinic acid treatment on the HSD-1 reductase activity of both doxycycline-treated and untreated tMSCs, and non-transfected MSCs. GA = 24h treatment of 10^−7^ M glycyrrhetinic acid. Dox = 72h treatment of 10 μg/ml doxycycline. Mann-Whitney *U* test, n = 4 for all groups, *p < 0.05, **p < 0.001, ns–non significant *p* > 0.05.

Non-transfected MSCs do not express functional HSD-1, even following treatment with doxycycline or pro-inflammatory mediators LPS and TNFα (Figure 2A). This indicates that in an inflammatory environment, any functional HSD-1 activity observed in tMSCs will only be due to expression of the HSD-1 transgene. We then showed that the HSD-1 expressed within tMSCs was functional as a reductase, able to convert cortisone into cortisol. We had minimal basal expression of construct independent of doxycycline, which is then significantly induced following 48 h of doxycycline treatment (Figure 2B). HSD-1 activity in tMSCs peaks after 48hrs exposure to doxycycline (median 8.23 pM/hour/100,000 cells), then plateaus with further exposure (Figure 2B). After 48 h culture with doxycycline, the tMSCs maintain constant levels of HSD-1 reductase activity for at least a further 72 h after removal of doxycycline from culture media (Figure 2C). This guided the preparation of tMSCs for future experiments, as use of doxycycline in co-culture experiments and models of lung injury would have a significant confounding factor due to its action as an antibiotic and matrix metalloproteinase inhibitor. Comparing with human alveolar macrophage HSD-1 activity data (Mahida et al., 2023), we found that the transgene-active tMSCs exhibit 41 times greater HSD-1 reductase activity than normal alveolar macrophages (8.23 vs. 0.2 pM/hour/100,000 cells). Reductase specificity to HSD-1 was assessed with glycyrrhetinic acid (GA), a selective HSD-1 inhibitor (Figure 2D). This resulted in a marked suppression of HSD-1 reductase activity in transfected MSCs. These data confirm that HSD-1 activity is markedly induced upon treatment with Dox in tMSCs within 48h relative to untreated tMSCs and MSCs.

### Impact of HSD-1 tMSC co-culture on human macrophage function

**FIGURE 3:**
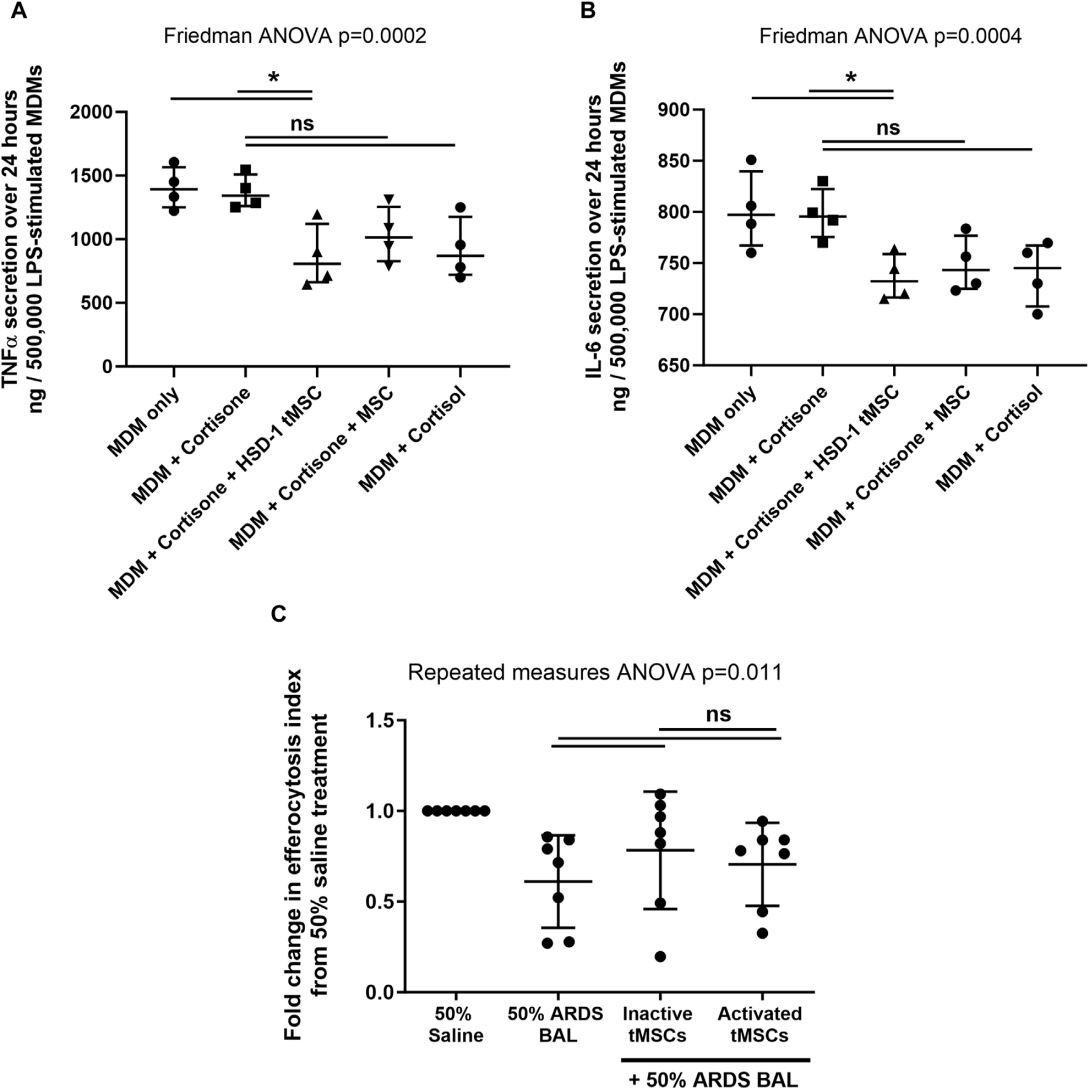
HSD-1 tMSCs co-culture with human macrophages **(A, B)**: MDMs were treated with LPS in the presence or absence of cortisone or cortisol (10^−7^ M). Cortisone-treated MDMs underwent direct co-culture with non-transfected MSCs or HSD-1 tMSCs for 24 h. MDM secretion of TNFα and IL-6 in conditioned media was then measured. Statistical analysis by Friedman ANOVA with Dunn’s multiple comparisons test, n = 4. **(C)**: Transwell co-culture of inactive tMSCs (mean of differences 0.173, *p* = 0.244) or activated tMSCs (mean of differences 0.095, *p* = 0.416) with 50% ARDS BALF-treated AMs had no significant effect on efferocytosis compared to ARDS BALF treatment alone. There is no significant difference in AM efferocytosis following inactive or active tMSC treatment (mean of differences 0.078, *p* = 0.50). *p < 0.05, **p < 0.001, ns–non significant *p* > 0.05.

To assess the functionality of doxycycline induced HSD-1 activity, we examined monocyte-derived macrophage cytokine output following LPS stimulation and co-culture with HSD-1 tMSCs and non-transfected MSCs in the presence of the inactive HSD-1 steroid substrate cortisone. To control for residual HSD1 activity in doxycycline-naive tMSCs, cells were pretreated with GA to minimise the underlying HSD-1 activity as a variable in steroid responses. Direct co-culture of transgene activated HSD-1 tMSCs with LPS-stimulated monocyte-derived macrophages (MDMs) from healthy volunteers (in the presence of cortisone) suppressed release of pro-inflammatory cytokines TNFα and IL-6, more effectively than culture with cortisone alone (Figures 3A, B).

Co-culture with non-transfected MSCs in the presence of cortisone, or with cortisol treatment alone, also showed a trend towards decrease in inflammatory cytokine release, however these did not reach significance (Figures 3A, B). There was no significant difference in inflammatory cytokine release following co-culture with HSD-1 tMSCs *versus* non-transfected MSCs (both in the presence of cortisone, Figures 3A, B). LPS treatment of non-transfected MSCs or transgene-activated HSD-1 tMSCs alone did not result in detectable TNFα or IL-6 secretion (data not shown). Untreated MDMs did not secrete detectable amounts of TNFα or IL-6 (data not shown).

We have previously developed an *in vitro* model of sepsis-related ARDS (Mahida et al., 2021b), in which primary human AMs from lobectomy patients are treated with pooled BAL fluid from patients with sepsis-related ARDS. The inflammatory cytokine content of pooled ARDS patient BAL fluid was characterised in our previous study (Mahida et al., 2021b). This treatment impaired AM efferocytosis and altered surface receptor expression, replicating functional defects observed *ex vivo* (Mahida et al., 2021a; Mahida et al., 2021b). We used this model to investigate whether co-culture with transgene-active HSD-1 tMSCs could restore AM efferocytosis and phenotype. In an *in vitro* model of sepsis-related ARDS, AM co-culture with transgene-inactive and transgene-active HSD-1 tMSCs showed a trend towards increased efferocytosis, however this did not reach significance (Figure 3C). In an *in vitro* model of sepsis-related ARDS, co-culture with transgene-inactive and transgene-active HSD-1 tMSCs had no impact on AM surface expression of C206, CD163, CD80, SIRPα or Mer (Supplementary Figure S4).

### HSD-1 transgenic MSCs reduce neutrophilic inflammation in a murine model of peritoneal sepsis

**FIGURE 4:**
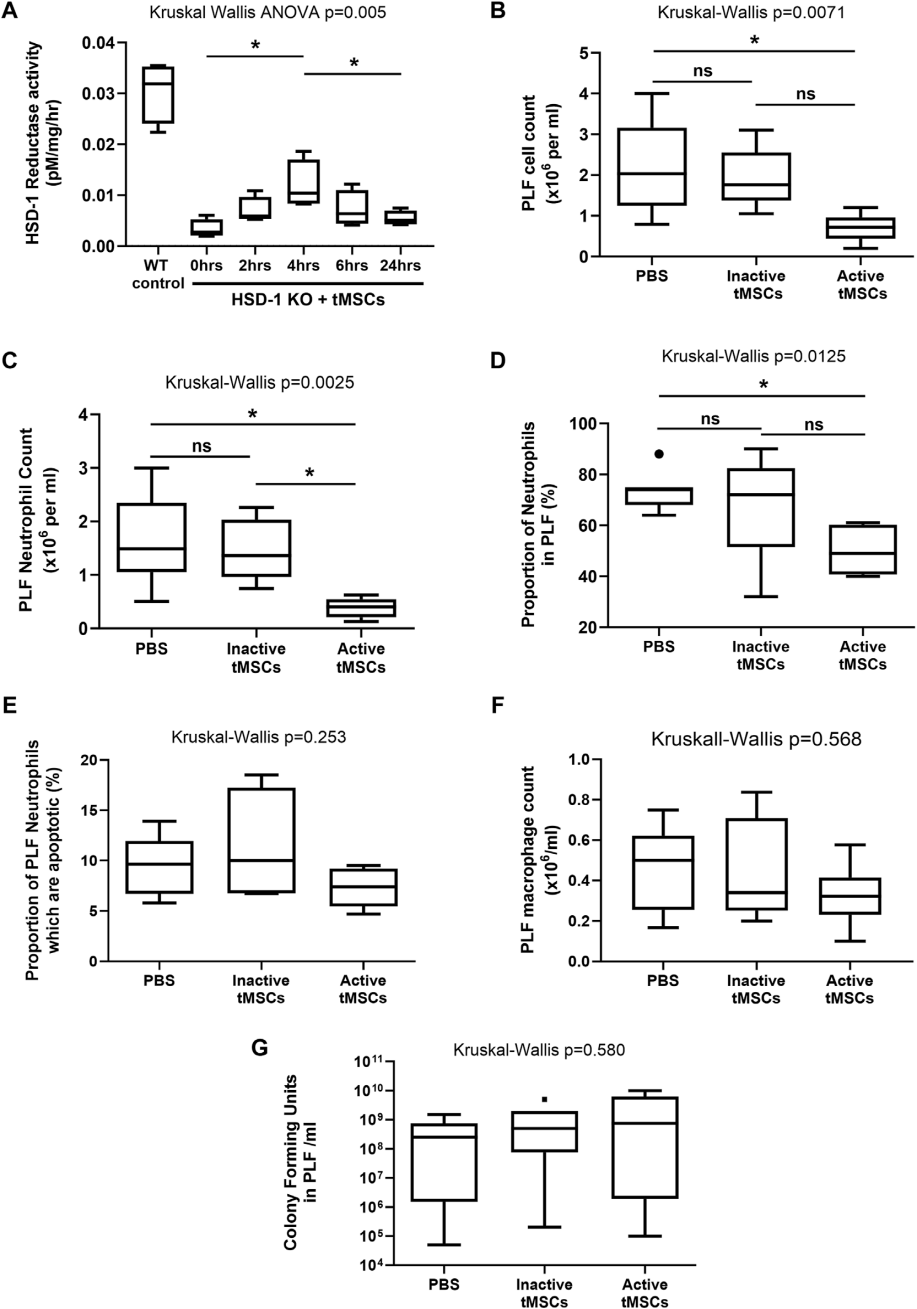
Peritoneal cellular recruitment and bacterial growth following a 3 h time course of CLP in WT mice. **(A)** a Time course of total lung HSD-1 reductase activity following tMSC administration in HSD-1 KO C57BL/6 mice was undertaken. 75,000 active HSD-1 tMSCs were administered intravenously to HSD-1 KO mice. “0 h” refers to HSD-KO mice which did not receive tMSCs. Untreated WT C57BL/6 mouse lungs were also analysed as a positive control. Statistical analysis by Mann Whitney test, n = 4 for all groups. **(B–F)**: WT C57BL/6 mice received PBS, 125,000 inactive tMSCs, or 125,000 active tMSCs intravenously 1 h prior to CLP. After 3 h, the PLF cell count **(B)**, PLF neutrophil count **(C)**, PLF neutrophil percentage **(D)**, PLF neutrophil apoptosis **(E)**, PLF macrophage count **(F)**, and PLF bacterial count **(G)** were measured. Data presented as Tukey box and whisker plots. Statistical analysis by Kruskal–Wallis tests and Dunn’s multiple comparison test, n = 6 for both groups. *p < 0.05, **p < 0.001, ns–non significant *p* > 0.05.

To assess HSD-1 activity following active tMSC administration, a time course was performed using uninjured HSD-1 KO C57BL/6J mice. The lungs were used as a surrogate location to measure HSD-1 activity over time, as MSCs are known to localise to the lung following intravenous administration (Rojas et al., 2005; Nemeth et al., 2009). This allowed us to determine the time between intravenous administration of tMSCs and maximal lung HSD-1 activity. Active HSD-1 tMSCs were treated with doxycycline *in vitro* for 48 h prior to removal of doxycycline and intravenous administration to mice. Lung HSD-1 reductase activity peaked at 4 h after intravenous administration of active tMSCs (Figure 4A, medians 0.0027 vs. 0.0104 pM/mg/hr, *p* = 0.029). After 4 h, lung HSD-1 reductase activity in HSD-1 KO mice was elevated to a level corresponding to approximately one-third of the activity seen in WT C57BL/6J mice (Figure 4A, medians 0.0104 vs. 0.0309 pM/mg/hr). Lung HSD-1 reductase activity 24 h after active tMSC administration was significantly reduced compared to activity seen at 4 h (Figure 4A, medians 0.0104 vs. 0.0051 pM/mg/hr, *p* = 0.029), and was not significantly different to lung HSD-1 activity in untreated HSD-1 KO mice (Figure 4A, medians 0.0027 vs. 0.0051 pM/mg/hr, *p* = 0.20). Therefore, using a shorter duration of the CLP model to 3 h (4 h post-tMSC administration) would allow us to assess the effect of peak HSD-1 transgene expression.

Wild Type (WT) C57BL/6J mice received either saline, 125,000 inactive HSD-1 tMSCs, or 125,000 active HSD-1 tMSCs intravenously 1 h prior to CLP. The endpoint was 3 h after CLP, and 4 h after tMSC administration, at the time when lung HSD-1 activity would be maximal. Treatment with active tMSCs significantly reduced PLF cell count compared to PBS (Figure 4B, medians 0.72 v 2.03 × 10^6^/ml, *p* = 0.024) but not inactive tMSC treatment (Figure 4B, medians 0.72 v 1.75 × 10^6^/ml, *p* = 0.059). Inactive tMSC treatment had no effect on PLF cell count compared to PBS treatment (Figure 4B, medians 1.75 v 2.03 × 10^6^/ml, *p* > 0.99). Treatment with active tMSCs significantly reduced PLF neutrophil count compared to PBS (Figure 4C, medians 0.403 v 1.49 × 10^6^/ml, *p* = 0.015) and inactive tMSC treatment (Figure 4C, medians 0.403 v 1.36 × 10^6^/ml, *p* = 0.033). Treatment with inactive tMSCs had no effect on PLF neutrophil count compared to PBS treatment (Figure 4C, medians 1.36 v 1.49 × 10^6^/ml, *p* > 0.99). Treatment with active tMSCs significantly reduced PLF neutrophil proportion compared to PBS (Figure 4D, medians 49% v 74%, *p* = 0.018) but not inactive tMSC treatment (Figure 4D, medians 49% v 72%, *p* = 0.154). Treatment with inactive tMSCs had no effect on PLF neutrophil proportion compared to PBS treatment (Figure 4D, medians 72% v 74%, *p* > 0.99). The treatment groups caused no significant difference in PLF neutrophil apoptosis (Figure 4E, *p* = 0.253), PLF macrophage count (Figure 4F, *p* = 0.568), or PLF bacterial count (Figure 4G, *p* = 0.580).

Following this 3 h CLP time course in WT mice, no significant differences were observed in the concentrations of PLF or serum cytokines between the three treatment groups: PBS, inactive tMSCs and active tMSCs (data not shown). Cytokines measured included MCP-1, MIP-1α, MIP-1β, MIP-2, KC, IL-1β, IL-6, IL-10, TNFα and VEGF.

## Discussion

In this study we generated and validated a recombinant lentivirus containing both the HSD-1 and GFP transgenes under the control of a tetracycline promoter. In subsequent transfection experiments we generated human MSCs containing this recombinant lentivirus to create HSD-1 tMSCs, with a transfection efficiency of 90.1%. Following exposure to doxycycline to induce transgene expression, the tMSCs co-expressed both HSD-1 and GFP protein with functional activity experiments confirming reductase activity and cortisol activation. We showed that the tMSCs retain a stem cell phenotype and possess the associated multipotency; the transfection process had not caused differentiation of the MSCs. Therefore, by retaining an MSC phenotype, the retained intrinsic anti-inflammatory capabilities of the tMSCs could combine synergistically with elevated HSD-1 transgene expression to reduce inflammation.

Transgene-activated HSD-1 tMSCs were able to partially suppress release of pro-inflammatory cytokines TNFα and IL-6 from LPS-stimulated MDMs. Albeit marginal, the co-culture results showed an additive anti-inflammatory effect from overexpression of the HSD-1 transgene in MSCs, compared to treatment with non-transfected MSCs or cortisol alone. However, the *in vitro* studies are simplified observations between two isolated cell types (neutrophils and macrophages). Although we saw reduced inflammatory cytokine production *in vitro* with HSD-1 tMSCs, this was not replicated *in vitro*. At the whole organism level, the complexity of interactions between multiple different cell types must be considered; additional cell types (including lymphocytes, natural killer and epithelial cells), which are not present in the *in vitro* model but still contribute to cytokine release, may in part contribute to the differences observed.

The inability of active HSD-1 tMSC co-culture to enhance the efferocytosis capacity of alveolar macrophages in an *in vitro* model of sepsis-related ARDS may indicate the presence of HSD-1 dependent and independent mechanisms of efferocytosis. The 6 h duration of co-culture may have been a limiting factor, being too brief to observe functional changes. However, with previous studies showing that MSC numbers in the lungs start to decrease by 3 h following intravenous administration, this model does reflect the duration that tMSCs would be present in the lungs if used as an intravenous clinical therapy (Rochefort et al., 2005). Further studies are required to determine whether multiple doses of HSD-1 tMSCs can influence macrophage efferocytosis.

Peritoneal sepsis remains a major cause of in-hospital mortality, with rates approaching 30% for patients admitted to the ICU due to the rapid onset of multi-organ failure (De Waele et al., 2014). The causes of peritoneal sepsis can include bowel perforation, colitis, malignancy, trauma, and abdominal surgery. Polymicrobial infection is common, especially in the context of faecal contamination. The mouse caecal ligation and puncture (CLP) model effectively replicates the polymicrobial infection and cellular inflammation observed in human peritoneal sepsis. Previous studies have found that intravenous administration of non-transgenic MSC in murine CLP results in increased survival, organ function, IL-10 secretion, bacterial clearance, monocyte phagocytosis and reduced inflammatory cytokine release (Gonzalez-Rey et al., 2009; Nemeth et al., 2009; Mei et al., 2010). We found that active HSD-1 tMSC treatment can reduce cellular inflammation in a murine model of peritoneal sepsis to a greater degree than inactive tMSCs. The ability of activated tMSCs to reduce neutrophilic infiltration in CLP compared to PBS and inactive tMSCs was observed at 3 h post-injury (4 h post-treatment). This timepoint was chosen maximal lung HSD-1 activity occurs 4 h after intravenous administration of HSD-1 tMSCs. This correlates with the findings of previous studies on MSC pharmacodynamics, which showed that MSC initially accumulate in the lungs following intravenous administration, however after 3 h their numbers in the lungs start to decrease (Rochefort et al., 2005; Lee et al., 2009; Eggenhofer et al., 2012; Leibacher and Henschler, 2016). This would account for the reduction in lung HSD-1 activity we observed after 4 h following HSD-1 tMSC administration. Previous studies in mouse CLP models showed that MSCs also accumulated in lymphoid tissues and the inflamed colon in the first day following administration (Gonzalez-Rey et al., 2009). Thus, HSD-1 tMSCs localise to the lungs and sites of inflammation systemically following administration.

HSD-1 expression in macrophages plays an important role mediating the anti-inflammatory effects of glucocorticoids at peripheral sites of inflammation. Our previous work has shown evidence of impaired macrophage HSD-1 autocrine glucocorticoid signalling in sepsis and sepsis-related ARDS, which contributes to the excessive inflammation observed (Mahida et al., 2023). We can infer that activated tMSC treatment restores HSD-1 reductase activity within inflamed tissues, leading to enhanced local activation of endogenous glucocorticoids, increased cortisol levels, and attenuation of the inflammatory response. Glucocorticoids also act on neutrophils to attenuate their activation, adhesion, margination, chemotaxis and accumulation in inflamed tissues (Ronchetti et al., 2018). Thus, the reduced neutrophilic infiltration observed after active HSD-1 tMSC treatment *in vivo* is thought to be due to the direct impact of elevated cortisol levels on neutrophils.

The potential therapeutic utility of HSD-1 tMSCs goes beyond that of sepsis; this intervention could show greater efficacy than MSCs alone in various inflammatory disorders including rheumatoid arthritis, sarcoidosis, and inflammatory bowel disease (McClain Caldwell et al., 2020; Lopez-Santalla et al., 2021; Dave et al., 2024). MSCs can induce an anti-inflammatory phenotype in alveolar macrophages isolated from sarcoidosis patients (McClain Caldwell et al., 2020). Preclinical studies in a Crohn’s disease model have shown that MSCs promote mucosal healing by reprogramming macrophages to an anti-inflammatory phenotype with enhanced efferocytosis capacity (Dave et al., 2024). The synergistic impact of upregulated HSD-1 reductase activity at the site(s) of inflammation may attenuate inflammatory injury to a greater degree in these disorders. Use of HSD-1 tMSCs could also act as an alternative to systemic exogenous glucocorticoid therapy in these disorders, to avoid the detrimental effects of long-term use. Thus, further studies investigating the administration of HSD-1 tMSCs in models of sterile inflammatory disease are required.

There are several limitations to this study. We were unable to analyse the fraction of M1 *versus* M2 macrophages in the PLF samples due to our staining panel, *ex vivo* macrophage efferocytosis was not assessed, and cortisol levels were not measured in the mouse studies. HSD-1 tMSC therapy was only assessed in a model of peritoneal sepsis. To fully model human sepsis, the intervention would also need to be assessed in a direct sepsis-related lung injury model (e.g., pneumonia). Administration of tMSCs prior to CLP injury does not reflect the clinical scenario of administrating treatment post-injury. Future studies would require tMSCs administration post-injury to assess treatment efficacy. Longer timescales will also be required to determine the impact of tMSCs on bacterial overgrowth. As HSD-1 transgene activity peaks at 4 h post tMSC administration, multiple doses of HSD-1 tMSCs may be required to maintain a constant HSD-1 transgene activity in future murine studies. Use of only male mice limits generalisability of the *in vivo* findings to both sexes.

In summary, we have created tMSCs which express functional HSD-1 enzyme, which can locally activate cortisol from the inactive precursor cortisone. HSD-1 tMSCs attenuated neutrophilic inflammation in a mouse CLP model and cytokine release by LPS-stimulated MDMs. Future studies investigating the anti-inflammatory capacity of HSD-1 tMSCs in models of sepsis-related direct lung injury and inflammatory diseases are required.

## Data Availability

The raw data supporting the conclusions of this article will be made available by the authors, without undue reservation.

## Abbreviations

APC, Allophycocyanin; ARDS, Acute respiratory distress syndrome; αMEM, α-Minimal essential media; DMEM, Dulbecco’s Modified Eagle’s Medium; FBS, Foetal bovine serum; GA, glycyrrhetinic acid; GFP, Green fluorescent protein; HEK, Human Embryonic Kidney cell; HSD-1, 11β hydroxysteroid dehydrogenase type-1; ISCT, International society for cellular therapy; LPS, Lipopolysaccaride; MOI, multiplicity of infection; (t)MSC, (Transgenic) Mesenchymal stem cell; PCR, polymerase chain reaction; SD, Standard deviation; TNFα, Tumour necrosis factor α.
