# Supplementary material for: 11β hydroxysteroid dehydrogenase type 1 transgenic mesenchymal stem cells attenuate inflammation in models of sepsis
Source: Front Bioeng Biotechnol. 2024 Jul 5;12:1422761. doi: 10.3389/fbioe.2024.1422761 (PMC11257926; doi:10.3389/fbioe.2024.1422761)
Supplement: Supplementary file 1 [file Table1.DOCX]

**Supplemental Information**

| **Anti-Human Antibody** | **Clone** | **Fluorophore** | **Manufacturer** | **Isotype Control** | **Concentration** |
| --- | --- | --- | --- | --- | --- |
| CD206 | 19.2 | APC | BD Biosciences | Mouse IgG1κ | 1 : 50 |
| CD80 | L307.4 | PE | BD Biosciences | Mouse IgG1κ | 1 : 50 |
| CD163 | GHI/61 | FITC | BD Biosciences | Mouse IgG1κ | 1 : 50 |
| MerTK | 125518 | APC | R&D Systems | Mouse IgG2B | 1 : 50 |
| SIRPα (CD163) | 15-414 | FITC | ThermoFisher | Mouse IgG1κ | 1 : 50 |

**Supplemental Table 1: Flow cytometry antibody staining panels to assess alveolar macrophage surface marker expression**

FITC: Fluorescein isothiocyanate. APC: Allophycocyanin. PE: Phycoerythrin. Mer: Mer Receptor Tyrosine Kinase. SIRPα: Signal-Regulatory Protein Alpha.

| **Antibody** | **Clone** | **Fluorophore** | **Manufacturer** | **Isotype Control** | **Dilution** |
| --- | --- | --- | --- | --- | --- |
| Anti-mouse F4/80 | T45-2342 | PE | BD Biosciences | Rat, IgG2Aκ | 1 : 100 |
| Anti-mouse CD11c | HL3 | PE-Cy7 | BD Biosciences | Hamster, IgG1 λ1 | 1 : 100 |
| Anti-mouse CD11b | M1/70 | APC | BD Biosciences | Rat, IgG2Bκ | 1 : 100 |
| Anti-mouse Ly6G (Gr1) | 1A8 | APC-Cy7 | BD Biosciences | Rat, IgG2Aκ | 1 : 100 |

**Supplemental Table 2: Flow cytometry antibody staining panel for murine peritoneal lavage fluid cells**

APC: Allophycocyanin. PE: Phycoerythrin.


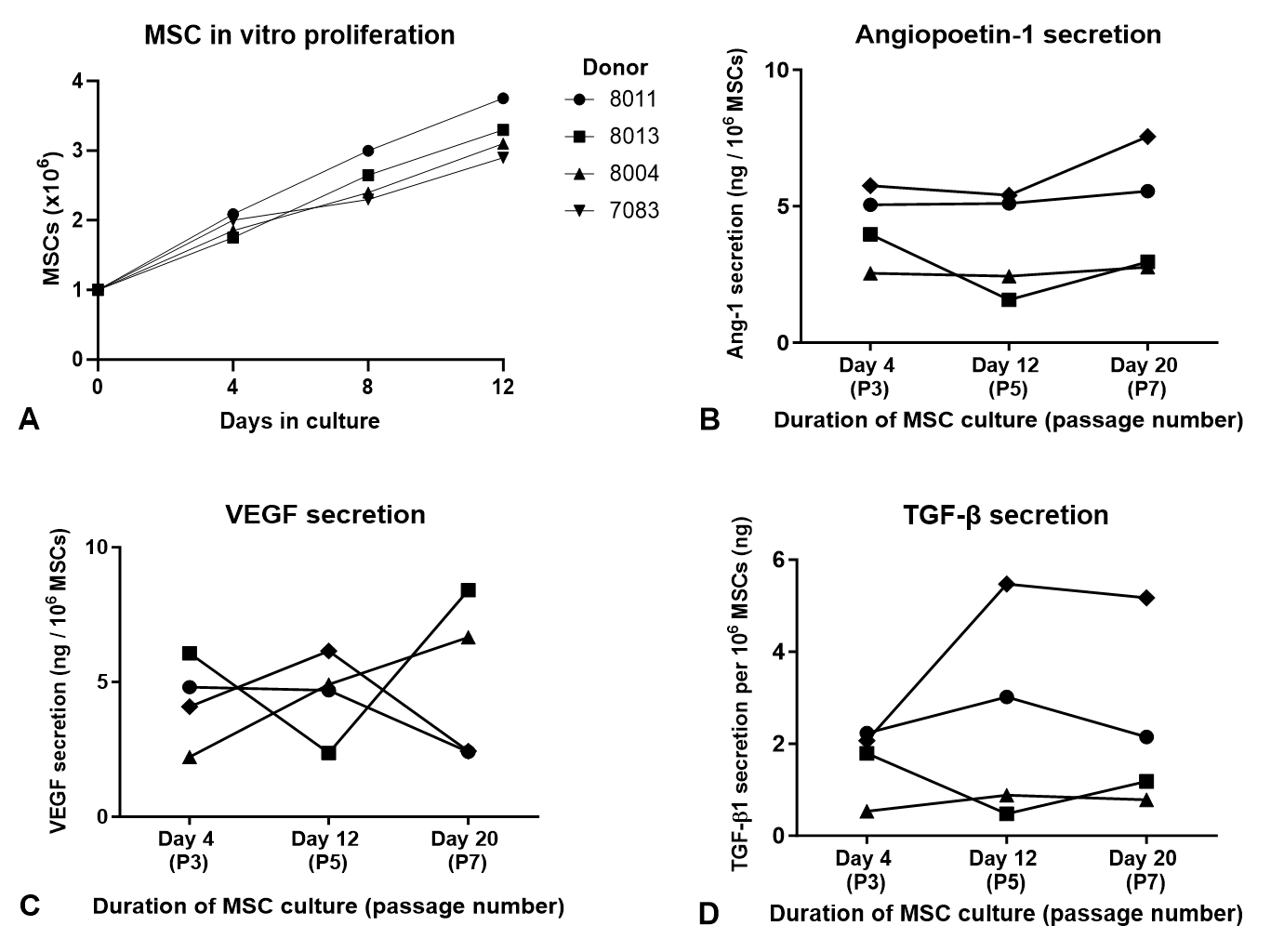


**Supplemental Figure 1: MSC proliferation and release of pro-resolving mediators following *in vitro* culture.**

**(A):** *In vitro* proliferation of human MSCs from 4 donors. MSCs from donor 8011 show the most rapid proliferation. **(B-D):** MSC release of pro-resolving mediators angiopoetin-1, VEGF and TGF-β. Variable release observed across 4 MSC donors.

**Supplemental Figure 2: Flow cytometry gating strategy for murine cell identification in peritoneal lavage fluid.**


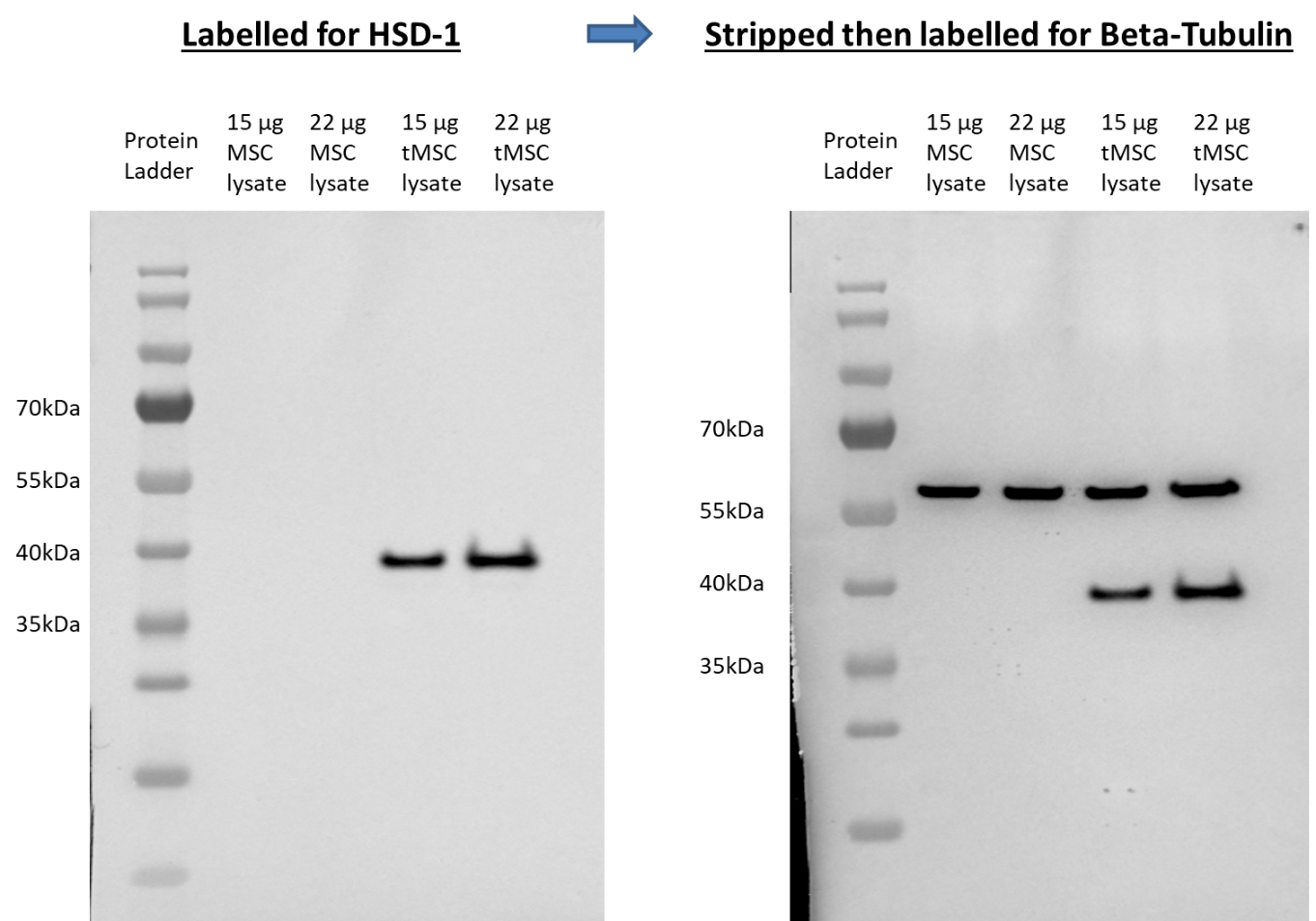


**Supplemental Figure 3: Western Blot showing HSD-1 (34kDa) and β-Tubulin (55kDa) protein expression in transfected MSCs**

Blots were initially labelled for HSD-1 (34 kDa), then stripped and labelled for β-Tubulin (55 kDa).


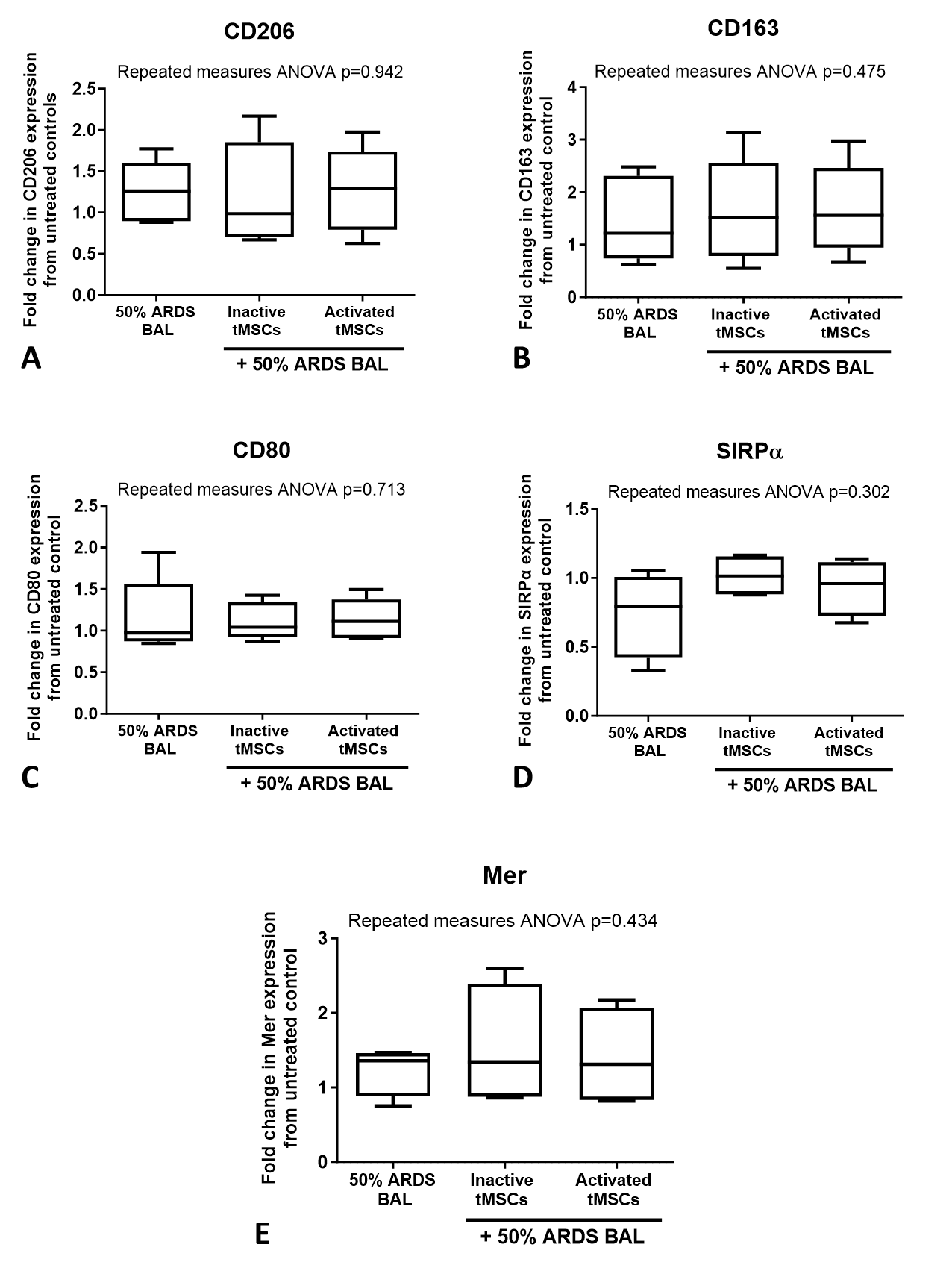


**Supplemental Figure 4: Effect of HSD-1 tMSCs on alveolar macrophage phenotype**

AMs were exposed to 50% ARDS BAL for 48 hours, then media was changed to serum-free RPMI containing 10-7M cortisone and a 6 hour transwell co-culture was performed with inactive HSD-1 tMSCs, activated HSD-1 tMSCs, or no cells. Following co-culture, surface marker expression was assessed. Statistical analysis by repeated measures ANOVA, n=4-5. A-F: Transwell co-culture of inactive or activated tMSCs with 50% ARDS BALF-treated AMs had no significant effect on surface expression of CD163, CD206, CD80, SIRPα or Mer compared to ARDS BALF treatment alone (repeated measures ANOVA p>0.30 for all).
